# Evaluating a peer-to-peer health education program in Australian public housing communities during the COVID-19 pandemic

**DOI:** 10.1186/s12913-024-10627-7

**Published:** 2024-02-27

**Authors:** Jane Oliver, Angeline Ferdinand, Awil Hussein, Ruqiyo Hussein, Jessica Kaufman, Peta Edler, Nicole Allard, Margie Danchin, Katherine B. Gibney

**Affiliations:** 1 Department of Infectious Diseases, University of Melbourne at the Peter Doherty Institute for Infection and Immunity, 792 Elizabeth St, Melbourne, VIC 3000 Australia; 2 Murdoch Children’s Research Institute, Melbourne, VIC 3052 Australia; 3 Centre for Health Policy, Melbourne School of Population and Global Health, University of Melbourne, Melbourne, VIC 3000 Australia; 4 North Melbourne Resident Action Group, Melbourne, VIC 3052 Australia; 5 cohealth, Melbourne, VIC 3066 Australia; 6 Department of Paediatrics, University of Melbourne, Melbourne, VIC 3000 Australia; 7 Department of General Medicine, The Royal Childrens Hospital Melbourne, Melbourne, VIC 3052 Australia

**Keywords:** COVID-19, Community health worker, Pandemic, Health services

## Abstract

**Background:**

The cohealth Health Concierge program operated in Melbourne, Australia from July 2020 to 30 June 2022. It provided peer-to-peer support to culturally and linguistically diverse residents of high-rise public housing. During this time, the COVID-19 public health response changed frequently and included movement restriction, testing and vaccination. We conducted a mixed-methods evaluation to determine the Health Concierge program’s impact on residents’ engagement with health services and public health activities.

**Methods:**

The evaluation, informed by a Project Reference Group, used the RE-AIM framework. We analysed data from 20,901 routinely collected forms describing interactions between Concierges and residents from August 2021 to May 2022. Additional evaluation-specific data were collected between March and May 2022 in four housing estates; we surveyed 301 residents and conducted 32 interviews with residents, Concierges and program stakeholders.

**Results:**

Concierges promoted COVID-safe behaviours; linked residents with support, testing and vaccination services; and disseminated up-to-date information. Of the 20,901 recorded interactions, 8,872 (42%) included Concierges providing support around COVID-19 vaccination. Most surveyed residents (191/301, 63%) reported speaking with a Concierge in the previous six months. The self-reported two-dose COVID-19 vaccine uptake was 94% (283/301). Some residents described having meaningful, appreciated conversations with Concierges, and some described superficial interactions. While residents initially welcomed the program, many felt it failed to evolve. Poorly defined management and hiring criteria led to variable program implementation. A need for bicultural workers to continue linking residents with services was discussed.

**Conclusions:**

Concierges’ impact on residents may have contributed to high community uptake of COVID-19 testing and vaccination, and had benefits beyond the COVID-19 remit. We recommend the program be revised and continued to inform further preparedness planning and support service access generally. Program models such as this have potential to inform and reassure high-risk communities during a pandemic. In addition, such programs can help overcome vaccine hesitancy and promote protective health behaviours, regardless of whether a pandemic is currently occurring. Ensuring these programs remain responsive to the changing needs of end-users needs over time is imperative.

**Supplementary Information:**

The online version contains supplementary material available at 10.1186/s12913-024-10627-7.

## Introduction

Community health workers (CHWs) have been employed in many settings to link communities with health systems and perform health promotion. While programs that engage CHWs to disseminate health information vary, evidence suggests that peer-based education may facilitate behavioural changes [1] and CHWs work can increase adherence to public health advice and medical treatment. Drawing on CHWs’ knowledge also may help clinical colleagues’ to improve their understanding of their patients [2]. The localized and place-based nature of many programs which involve CHWs disseminating health information is often critical for improving health outcomes [3, 4]. 

CHWs have been reported to be active in public housing communities in Victoria, Australia [5, 6]. During the 1960s, the Housing Commission of Victoria organised construction of high-rise residential estates for public housing tenants across 32 sites in 19 suburbs of Melbourne [7]. Communities at these sites are culturally and linguistically diverse (CALD), with many residents (or their parents) born in low/middle-income countries and moving to Australia as refugees [8]. Residents of these estates were disproportionately impacted by the COVID-19 pandemic [9]. Prior to July 2020, widespread transmission of SARS-CoV-2 had been avoided in Melbourne. In early July 2020, detection of 23 COVID-19 cases in Melbourne public housing estates prompted fears of rapid transmission [10]. In response, on 4 July 2020 ‘Operation Benessere‘ commenced [9, 11], with approximately 3,000 residents of estates in two suburbs - Flemington and North Melbourne– placed in ‘hard lockdown’. With no advance warning, these residents were detained in their apartments by order of the Victorian Government for a period of 5–14 days. This restriction was in addition to COVID-19-related public health restrictions applied throughout Melbourne, including among the world’s longest cumulative periods spent in lockdown during 2020–2021 [9]. In December 2020 the Victorian Ombudsman concluded Operation Benessere breached residents’ human rights [9]. In September 2020, following Operation Benessere, the Victorian Government initiated the High Risk Accommodation Response (HRAR) in public housing and other high-risk accommodation settings [12, 13]. HRAR was a partnership between 24 lead community healthcare provider organisations and the Victorian Government [14]. Outreach services were provided to residents, many of whom had unmet health needs and little prior contact with support services [15]. HRAR’s core functions included catchment planning, community engagement, prevention and preparedness activities, and supporting COVID-19 outbreak responses. Over time, HRAR evolved to include supporting residents’ access to COVID-19 testing and vaccination services [16]. cohealth, one of Victoria’s largest community providers [17], provided health and wellbeing services to residents undergoing Operation Benessere [9] and later provided additional services to residents in public housing as a HRAR lead provider [6]. Key dates relevant to this evaluation period are listed in Supplementary Fig. 1.

The cohealth Health Concierge program (‘the program’) was designed to provide place-based peer-to-peer COVID-19 education to residents of high-rise estates as part of HRAR [5, 6]. Concierges were stationed in foyers of 31 residential high-rises in eight Melbourne suburbs. Their role was to share information about public health restrictions, COVID-19 and health services (including testing and vaccination), and act as community supports. A major focus was providing accurate, up-to-date, understandable COVID-19 information following a morning briefing with the onsite cohealth nurse manager [5, 6]. cohealth reportedly employed more than 150 Concierges, the majority from bicultural and multilingual backgrounds. Many Concierges were public housing residents– some dwelled in the estate where they worked [18]. cohealth’s Concierge training included short courses in COVID-19 safety, de-escalation and emotional intelligence. In addition, daily de-briefing opportunities with the on-site nurse and Client Services Officer were offered [18]. Concierges worked alongside cohealth health and support staff [19]. Service hours varied; at most with Concierge stations staffed 7-days per week from 9am until 6pm [20]. Concierges also helped organise community wellbeing activities [21]. Prior to our evaluation, media reports indicated the program was promoting COVID-19-safe behaviours including COVID-19 vaccine uptake [22, 23]. Concierges themselves were said to have benefited from their work [5, 19]. In March 2022, the Victorian Healthcare Association (the peak body representing the public health sector and supporting Victoria’s public health services) proposed embedding the HRAR model of care within the Victorian public health system [24]. However, pandemic response funding and the Concierge Program were discontinued from 30 June 2022, prior to transitioning to a new program (called ‘Community Connectors’) and a ‘COVID-normal way of life.’ [15].

To assess the impact of the program on high-rise public housing residents’ engagement with health services and public health activities, we conducted a mixed-methods evaluation. This evaluation was planned and initiated prior to the discontinuation of the program.

## Methods

We used a co-design evaluation methodology based around the Reach, Effectiveness, Adoption, Implementation, and Maintenance (RE-AIM) framework, and the consolidated criteria for reporting qualitative research (COREQ) framework for reporting [25, 26]. A Project Reference Group (PRG), including residents, Concierges, other cohealth staff and Victorian Government Department stakeholders guided all study processes including the study design, results interpretation and recommendations made. Each PRG session was structured to prioritise residents’ opinions being heard.

### Setting

This evaluation was undertaken in from March to May 2022 in four distinct high-rise public housing estates with large populations and where the program was well established: North Melbourne and Flemington (exposed to Operation Benessere), and Carlton and Collingwood (selected due to having comparable population sizes but were not directly impacted by Operation Benessere). These four estates were selected to investigate whether residents’ trust scores differed according to whether their estate was exposed to Operation Benessere. Supplementary Fig. 2 displays locations of the included estates.

In March 2022, each included estate housed between 1,295 and 1,965 residents. More than half the residents in each estate were reported to have CALD backgrounds, with Somalia, Ethiopia, Vietnam and China the most common countries of birth after Australia *(Source: Private communication from the Victorian Government Department of Families, Fairness and Housing (DFFH) addressed to Jane Oliver; 1 July 2022)*.

### RE-AIM indicators and data sources

#### Health concierge interaction forms

cohealth required Concierges to record each interaction with residents using an Interaction Form (Appendix 1). These routinely collected data contributed to the reach and effectiveness aspects of the evaluation, and included the date of the interaction, the location, and the type of supports the Concierge provided. Residents provided scores from 1 to 10 (10 being highest) for how useful they found the Concierge’s service, and how likely they were to recommend the service to friends or family. These scores were reported to the Concierge, who recorded it. cohealth provided Interaction Form data from included estates for analysis, covering 1 August 2021 to 12 May 2022 (9.5 months).

#### Surveys

A survey co-designed with the PRG comprised 35 short-answer/multiple choice questions (Appendix 2) contributing data on program reach, effectiveness and adoption. It assessed Concierge competence, Concierge respectful communication and trust in public health authorities. Questions drew on two standard questionnaires [27, 28] adapted to refer to Concierges and an infectious disease outbreak. Participants were asked to self-report the number of COVID-19 vaccines and COVID-19 tests they had ever had, and their trust in COVID-19 vaccines (assessed using Likert scales). We aimed to survey approximately 75 residents aged > 15 years in each of the four included estates. This sample size was selected to provide 80% power to detect an exposure difference of 11% between estates that had, and had not, been exposed to Operation Benessere.

Five Research Assistants administered the survey to residents using iPads. They were trained in data collection the week prior and had never worked as Concierges or cohealth employees. Three were residents of Flemington and North Melbourne estates, however they did not collect data within their estate. The other two did not currently live in public housing, however they were familiar with the study sites through lived experience. Between them, the Research Assistants were fluent in English, Somali, Arabic, Malay and Vietnamese. Convenience sampling in shared community areas occurred during weekdays (at Carlton, Collingwood and North Melbourne) and weekends (in Collingwood and Flemington). Research Assistants approached potential participants using a language they thought the person might understand. If interest was indicated, they explained the evaluation and survey questions to residents using English/other languages, as appropriate. Sometimes residents chose to act as interpreters enabling other residents to compete the survey when they could not otherwise communicate with the research team. Surveying was performed from 16 March to 2 April 2022. Participants were thanked with a $20 gift voucher.

#### Interviews

Semi-structed interview guides were developed by qualitative researchers within the Evaluation Team and refined with advice from the PRG (Table 1, Appendices 3–6). Interviews contributed data on all indicators of the RE-AIM framework. Residents, Concierges, other cohealth staff and other stakeholders were interviewed (Table 1). ‘Other stakeholders’ worked with residents or Concierges but were not cohealth employees or residents, and included staff from Foundation House, a not-for-profit residential rehabilitation centre which provided support services to Concierges.

**Table 1:** Participant groups interviewed from 1 April to 23 May 2022

Participant group	Identification	Recruitment	Interview topics
Residents	Responded ‘Yes’ in survey to being contacted about future research and provided contact information	Email/SMS invitation	Feelings about interacting with Concierges. Whether they thought Concierges were helpful. How their estates’ needs might be met effectively.
Concierges	Identified by the Evaluation Team / Project Reference Group / cohealth staff as possibly being interested	Email invitation	The type of work they did as Concierges. Perceptions of their usefulness to residents.
Other cohealth staff	Identified by the Evaluation Team / Project Reference Group as possibly being interested	Email invitation	Participants’ work with Concierges. Perceptions of Concierges usefulness to residents. cohealth’s responsiveness to residents’ health and wellbeing needs. How residents’ health and wellbeing needs might be met effectively by the Concierge program.
Other stakeholders			

Participants spoke until they indicated they had nothing to add. Interviews could be conducted in any language with the interviewer translating and explaining as necessary with audio recording enabled. No interview participants were known to the interviewers. No others were present during the interviews. Participants were interviewed once.

### Analysis

Interaction Form data underwent simple descriptive analyses.

For survey data, median scores for Concierge Competence, Concierge Respectful Communication, and Trust in Public Health Authorities were calculated by converting all responses in each survey category to a score between 0 and 3, then taking the mean value for each category. Questions were weighted equally. The Mann Whitney U test was used to compare median scores between estates. Multivariate ordinal regression estimated the adjusted odds ratio (aOR) with 95% confidence intervals (CIs) of an increased category score, in order to identify any predictor variables. Possible predictor variables considered were: participant demographics; number of COVID-19 tests; number of COVID-19 vaccines; and whether the estate had experienced hard lockdown. This enabled subgroups with higher and lower scores to be identified. Results were considered statistically significant where *p* < 0.05. The proportional odds assumptions were tested by undertaking a series of binary logistic regression models (with the outcome dichotomised e.g. by comparing scores of < 5 with scores of 5 or more) and ensuring the coefficients for each covariate were similar.

Interview audio recordings were de-identified and transcribed. The interviewer transcribed the Somali interviews and translated the transcript to English. JO performed an initial inductive descriptive thematic analysis. After reviewing the transcripts, JO categorised data into codes and sub-codes, then grouped codes thematically using NVivo version 12 plus [29]. JO used a virtual whiteboard (miro.com) to identify common and unique themes and sub-themes, then refined and named themes with input from the study team and the PRG. The PRG provided direction around contextualising the themes and making recommendations that would help to meet the residents’ needs. Final themes were organised deductively within the RE-AIM framework [25]. Quotes are provided to support the thematic analysis, some modified slightly to assist readability.

## Results

We obtained 20,901 Interaction Forms (routinely collected August 2021–May 2022), surveyed 301 residents (March–April 2022), and interviewed 32 stakeholders (April–May 2022).

The majority of surveyed participants were female (*n* = 212; 70.4%). Many (*n* = 249; 82.7%) mostly spoke languages other than English at home, most often Somali (37.9%; *n* = 114). Eleven participants (3.7%) were Aboriginal and/or Torres Strait Islanders. The median age was 45 years (range 16–101 years; Table 2).

**Table 2:** Demographic characteristics of survey participants

	**Participants [N = 301]**	
	*n*	%
**Gender**		
Female	212	70.4
Male	89	29.6
Other/Prefer not to say	0	0.0
**Age, years**		
Median 45 years; range 16–101 years; interquartile range (IQR) 32–60 years		
**Language mostly spoken at home**		
Somali	114	37.9
English	52	17.3
Arabic	38	12.6
Vietnamese	26	8.6
Tigrinya	15	5
Other (N = 23 languages)	54	17.9
Blank	2	0.7
**No. people living in household**		
Median 3 people; range 1–11 people; IQR 2–5 people		
**Aboriginal Australian/Torres Strait Islander**	11	3.7
**Residential estate**		
Carlton	74	24.6
Collingwood	77	25.6
Flemington	76	25.2
North Melbourne	74	24.6

Interviewed stakeholders comprised 14 residents (2–5 from each estate); eight Concierges (1–3 from each estate), five other cohealth staff; three DFFH staff and two Foundation House staff. Interviews with residents tended to be briefer than with other participants (mean duration 17 min versus 41 min). Thirteen of 14 interviewed residents mostly spoke a language other than English at home: seven spoke Somali, three Arabic, and one each of Cantonese, Hindi and Tigrigna. As 37 residents were invited, the resident interview response rate was 38%, with 23 not responding (none explicitly declined). No participants chose to review their interview transcripts. Two face-to-face interviews were conducted in Somali by Research Assistants who resided in included estates (RH; female, Bachelor of Applied Science, no prior qualitative research experience. SA; female, no tertiary qualifications or prior qualitative research experience). They had received two training sessions with JO (Research Fellow). Due to their limited availability, JO (female, PhD in public health, qualitative research experience) conducted all other interviews in English; six were face-to-face; 11 residents were interviewed by phone and 10 cohealth staff / other stakeholder interviews were via Zoom.

Key themes are presented according to the dimensions of the RE-AIM framework and related study outcome (Table 3).

**Table 3:** Dimensions of the RE-AIM framework and relationship to thematic findings and study outcomes*

RE-AIM framework dimension and key considerations	Application to the thematic analysis	Key theme
**Reach** into the target population. Who benefited from the intervention?	Did the program reach the residents? Did it reach those most in need?	*Everyone knows they’re there* By stationing Concierges in residential building foyers, residents frequently encountered Concierges and had ready access to the information Concierges offered.
**Effectiveness** How favourably did the intervention perform in practice?	Did the program achieve its goals?^	*Concierges were great at the start of the pandemic, they don’t do so much now* While the program was valued during the initial rapidly changing public health response, many felt it had failed to evolve in the ‘COVID-normal’ phase. While some residents described having meaningful conversations with Concierges, many interactions described were superficial. Some felt the true impact of Concierges’ work was underrecognized.
**Adoption** considering target settings, institutions and staff.	To what extent did residents engage with Concierges?	*Inconsistent program adoption within communities* Perceptions around whether Concierges helped the community differed considerably. Some residents described feeling little connection with Concierges, while others valued Concierges and considered them part of their community.
**Implementation** considering the consistency and cost of delivering the intervention.	How consistently was the program delivered by Concierges?	*Unclear expectations led to variable service* A lack of clarity around what the Concierge role required was a barrier to effective service. The need to employ people who actually engaged with, and represented, the people they served came up frequently.
**Maintenance** of intervention effects over time. Did the intervention produce desirable outcomes? How can this be sustained?	To what extent did the program become part of routine practice and maintain effectiveness?	*There’s so much more we could do* Many people mentioned an ongoing need for a bicultural information hub in the estates. There was a perception that not continuing the Concierge program was a missed opportunity to deliver health promotion and link residents with services.

*Table adapted from Foreman et al., 2017 [30].

^Program goals: To share information about COVID-19, public health restrictions, health services, and have Concierges act as community supports [5, 6].

### Reach: everyone knows they’re there

Three-quarters of surveyed residents (225/301, 75%) reported ever having received information about testing from a Concierge, 92.0% of whom (207/225) reported they thought this information was accurate. Most surveyed residents (88%; 266/301) reported they had been tested for COVID-19 at least once, and 30% (91/301) reported being tested more than five times. A similar proportion (73%; 219/301) reported ever having received information about COVID-19 vaccines from a Concierge, 93% (203/219) of whom thought this information was accurate. Two-thirds, (191/301, 63%) reported speaking to a Concierge in their building in the prior six months; 14% (42/301) spoke to a Concierge more than five times in that period.

When interviewed, residents immediately recalled seeing Concierges and understood their role was to provide COVID-19 information and facemasks. Some remarked on seeing Concierges often and when asked, most said they would feel comfortable talking with a Concierge.

*“I’ve seen [Concierges] a lot. I would just go up to them, especially because they are under my building, like “hey is it okay if I have face masks?”* Resident #7, Carlton.

Interaction Form data showed Concierges provided COVID-19 related support in all 20,901 recorded interactions. In addition, ‘other’ support was provided in 78.3% (*N* = 16,365) of interactions, most commonly ‘Other health-related information’ (Table 4). On average, 5.5 supports were provided per interaction (range: 1–8 supports/interaction). Language support was provided in 8.7% (*N* = 2,359) of interactions.

**Table 4:** Supports recorded in Interaction Forms from Carlton, Collingwood, Flemington and North Melbourne estates, 1 August 2021 to 12 May 2022*

Type of support	No. of supports	% of supports [N = 115,753]	% of interactions [N = 20,901]
**COVID-19-related supports**			
COVID-19 related information	11,726	10.1	56.1
Booking a COVID-19 test	1,068	0.9	5.1
Support to get tested	10,191	8.9	47.9
Booking a COVID-19 vaccine appointment	606	0.5	2.9
Support to get vaccinated	8,872	7.7	42.4
Face mask distribution	19,999	17.3	95.7
Hand sanitiser distribution	17,281	14.9	82.7
Well-being / Social check-in	13,879	12	66.4
**COVID-19-related support total**	**83,448**	**72.1**	**100**
**Other supports**			
Other health related information	13,785	11.9	66
Mental health information	5,220	4.5	25
Housing support	4,856	4.2	23.2
Financial support information	4,003	3.5	19.2
Education support information	3,797	3.3	18.2
**Other support total**	**32,305**	**27.9**	**78.3**

* Data collected routinely by Concierges during the program

**Fig. 1:**
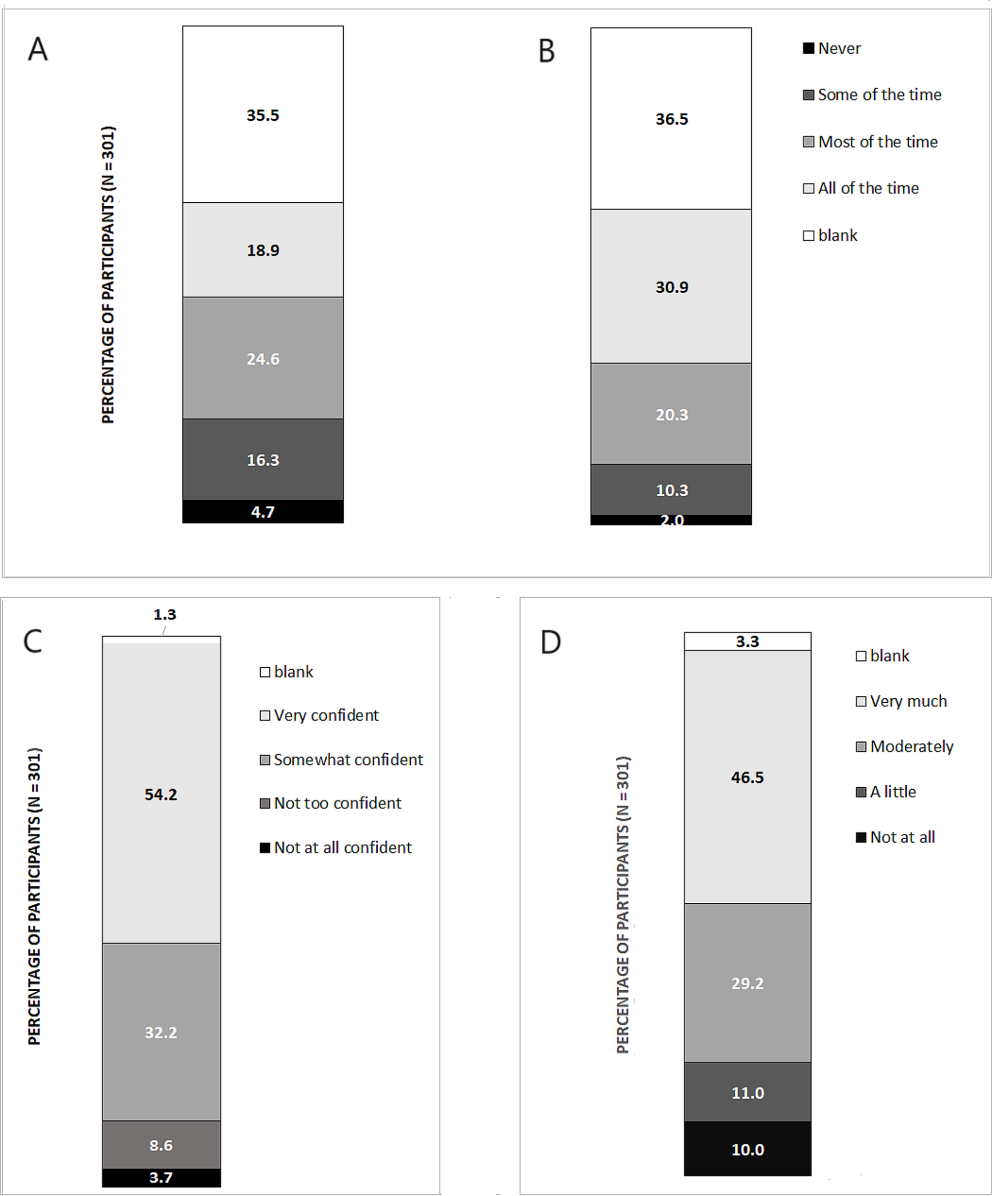
Likert scores assigned by survey participants– (**A**) Concierge healthcare competence, (**B**) Concierge respectful communication, (**C**) Trust in Victorian public health authorities, (**D**) Trust in COVID-19 vaccines

Nearly all (96.0%; *n* = 289/301) surveyed residents reported having received at least one COVID-19 vaccine, with 94.0% (*n* = 283) reported they had completed the recommended two-dose primary vaccine course. Nearly half (*n* = 39, 46.2%) reported they had received a booster (third) dose, lower than the proportion of eligible Victorians who had received a booster (65.9% as of 17 March 2022; all participants were eligible) [31]. Trust in COVID-19 vaccines was fairly high; 75.7% (*n* = 228) stated they moderately/very much trusted COVID-19 vaccines (Fig. 1).

### Effectiveness: concierges were great at the start of the pandemic, they don’t do so much now

The median score from the Interaction Form data regarding satisfaction with the Concierge Service was 10 out of 10 (range: 1–10; IQR: 10–10), although these scores may have been affected by response bias. Residents shared experiences of Concierges supporting them whilst in home isolation, bringing supplies and keeping them updated on COVID-19-related news. Interviewed residents often identified Concierges as an important source of up-to-date information when public health advice and restrictions were rapidly changing. A few residents described Concierges teaching them ways to protect themselves from COVID-19 and being reminded to remain vigilant upon seeing them. One resident shared discussing COVID-19 vaccination with a Concierge.

*‘Because of the information they give me, that lead me to take the vaccination. To protect myself and my children.… I’m immunocompromised.’ Resident #10, Collingwood*.

Generally, however, residents expressed there was no ongoing need for Concierges to be stationed in foyers sharing COVID-19 information once the public health response moved away from tight suppression approaches.

When the pandemic start, that’s when I feel like they [Concierges] were more useful… now, they just sit there. Resident #1, North Melbourne.

Some Concierges disagreed, saying that residents came to them for reassurance as COVID-19 case numbers increased. While Concierges described working hard to support residents, some said they felt restricted behind their desks and wanted to engage more with the community.

Superficial interactions with Concierges while obtaining face masks and hand sanitizer were commonly described by residents, especially as pandemic restrictions eased. Residents often described Concierges as a source of simple, practical COVID-19 advice, such as where to get a COVID-19 vaccine. Residents largely perceived Concierges as having limited education and training, and therefore not being able to answer technical questions, which they would save for a medical provider. Most residents said they would be unwilling to discuss non-COVID-19 health matters with Concierges.

*“I didn’t ask them [Concierges] much questions, I just wanted to know where I could get the vaccination and they told me… I feel like they are not like educated… the doctor would know more.” Resident #1, North Melbourne*.

The median Concierge Health Care Competence Score was 2 out of 3 (IQR:1–3), equivalent to surveyed residents reporting the service was provided competently most of the time. The multivariate ordinal regression model indicated that residents who had > 5 COVID-19 tests had higher odds of giving a higher Competence Score compared to residents who had ≤ 5 COVID-19 tests (aOR: 2.47; 95% CI: 1.35–4.51; *p*-value: 0.003; Supplementary Table 1).

The median Respectful Communication Score was 3 out of 3 (IQR:1–3), equivalent to residents reporting Concierges communicated respectfully all the time. The proportion of participants assigning different score categories is shown in Fig. 1. No predictors of a higher Respectful Communication Score were identified by the model.

A subtheme concerned under-recognition of the full impact of Concierges’ work. cohealth staff discussed Concierges’ organising community services and events, including clothing libraries, walking groups and festivals. Some suggested the residents simply saw these as being cohealth-run and were unaware of the Concierges’ contributions.

cohealth staff discussed Concierges’ insights as critical to informing their health service; particularly mass COVID-19 vaccination and testing services.

*“[Concierges]….were saying, “can you change your operating hours to 12 to 6:30, so you can capture everyone coming home?” “Can we make sure we have got these languages on this day?” cohealth staff #1*.

Several cohealth staff felt that the program was constrained by its COVID-19 funding remit, and the impact of Concierges work was not captured by HRAR indicators, nor recognised by funders.

*“…we [cohealth] will continue to step into some other issues that are onsite like mental health.…[the program] it’s been labelled COVID response.…it’s more than that.” cohealth staff #5*.

### Adoption: inconsistent program adoption within communities

No interviewed residents said that a Concierge would be their first source of COVID-19 advice; most would initially look at Health Department webpages. Despite this, some, including those who had very limited interactions with Concierges, said they felt the program helped residents and it was a shame it would not continue. cohealth staff and Concierges emphasised the close, trusted relationships which they perceived Concierges had built with residents, and spoke of how this trust carried through to other cohealth services. Conversely several residents and Concierges discussed residents being too busy to speak with Concierges, and some residents described feeling people in their high-rises had very little connection to Concierges.

*‘They [Concierges] are just sitting at the bottom at the door and people are going and coming… after some time, people just started ignoring them.…They didn’t even connect with the community around here.’ Resident #2, North Melbourne*.

Some residents strongly criticized Concierges for their perceived lack of engagement. They said the estate resident-leaders had to step in to support vulnerable neighbours through the pandemic and promote COVID-19 vaccine uptake themselves as no-one else was.

*“The Concierge is… there with the paper [COVID-19 vaccine pamphlet], but the African communities [residents], they are not believing the paper. Some people can’t read… But as a community leader,… we mention how easy and important [COVID-19 vaccination] is… The community leaders, they are working hard to get the community to do it, but I don’t know about the Concierge.” Resident #11, Flemington*.

Meanwhile, other residents, Concierges and other cohealth staff described Concierges providing highly varied, impactful support to residents, not limited to COVID-19/related issues.

*“[Concierges] They’re friendly people. And they’re working very hard. They will give you information. They are so nice…. They understand what the community needs. They are part of the community too.” Resident #3, Collingwood*.

Surveyed residents scored their trust in the Victorian public health authorities’ ability to respond to a localised infectious disease outbreak highly. The median confidence score was 3 (‘very confident’); range 0–3; IQR: 2–3. No difference in the median score was observed according to whether or not estates experienced Operation Benessere (*p* = 0.52). Participants who mostly spoke languages other than English at home scored higher (aOR: 2.74 [95% CI: 1.49–5.07]; *p*-value: 0.001), as did participants who reported having ≥ 2 COVID-19 vaccine doses compared to participants who had < 2 COVID-19 vaccine doses (aOR: 5.27 [95%CI: 1.89–14.7]; *p*-value: 0.0002; Supplementary Table 1).

### Implementation: unclear expectations led to variable service

Poorly defined hiring criteria were sometimes said by interviewed participants to have resulted in inappropriate people becoming Concierges. The merits of employing residents were often discussed. A need for this to occur in a meaningful way, employing people who actually engaged with, and represented, the community they served came up frequently. Concierges sometimes discussed a lack of clarity around their management and reporting pathways, inadequate supervision and unclear expectations around what they were required to do. This confusion was said to have caused some Concierges to disengage, feel bored and behave unprofessionally.

*“…we had management around sometimes, but generally we were just left to our own devices. That was pretty ugly. And it meant also that the Concierges who were always naughty got even naughtier.” Concierge #7*.

Some Concierges felt the training they had received was too little, too late. This view was also articulated by a Foundation House employee.

*‘I think by 2021, [ Concierges ] were more confident in the role but… there didn’t really seem to be much change in the support available for them, especially around risky situations [aggression from residents].…but people still kept loving the job.’ Other stakeholder #5*.

### Maintenance: there’s so much more we could do

cohealth staff and Concierges commonly voiced a perception that not expanding the program was a missed opportunity. Interviewed people, including residents, discussed a need for a community health hub, or similar service, where bicultural workers could provide residents with referrals to varied service providers, conduct health promotion and act as an information point. Residents spoke of their estates having building maintenance issues, residents needing language support, experiencing food insecurity, and requiring greater access to medical, oral and mental health services. Some discussed how Concierges helped to address these issues.

…he [a Concierge] thought it would be really important to create a space, initially where men could talk about mental health, so he created a wellness walk, which we do on most Tuesday and Thursday mornings and now it’s sort of open to everybody.

cohealth staff #3

Interviewed concierges spoke of the sense of purpose their work brought them and how much they enjoyed helping residents. This came despite more difficult aspects of the role, particularly having to work in uncomfortable temperatures and sometimes facing aggression. Several single mothers described how working flexible hours on-site allowed them to have employment despite their caring responsibilities. Concierges expressed grief, anger and worry that their roles were to soon end abruptly. They described feeling unappreciated and used by government, and also by cohealth. They discussed their perceptions that their work benefitted end-users and there was a need for it to continue. Concierges shared sacrifices they had made for their work, the emotional toll of working at the estate they lived in, and their dedication to helping others.

*[Said through tears] ‘Upset, worried. Very upset with how it is and we help the community a lot. Why is the job gone? And we were working very hard for this government.’ Concierge #6*.

## Discussion

### Key findings

The program had an extensive reach, with 20,901 interactions recorded over 9.5-months and nearly two-thirds of surveyed residents having spoken with a Concierge in the last six months. Through being stationed in residential high-rises with on-site health workers and support staff, Concierges could rapidly update residents, provide reassurance and support outbreak investigations. This was despite strict public health restrictions frequently limiting in-person interactions [32]. Most surveyed residents (73–75%) reported obtaining information from a Concierge about COVID-19 vaccines and testing, and Interaction Forms indicated the Concierges provided very frequent support to get tested and vaccinated (53% and 45% of interactions, respectively). Our surveys indicate the Concierge service was mostly delivered competently and Concierges communicated respectfully. There was a perception that the program became less effective, with the scope needing to be modified repeatedly to meet residents’ needs as pandemic restrictions eased. Some participants criticised Concierges over a perceived lack of engagement, especially once most pandemic restrictions had lifted. Others said Concierges helped the residents. There was some tension between residents seeing Concierges as approachable peers, yet perceiving them as unable to answer technical questions, which they would save for medical professionals. Variable implementation was apparent, with management described as inconsistent across sites, and Concierge personnel not necessarily representing the residents they served. A need to maintain a revised program that sought to address outcomes beyond COVID-19 was often discussed in the context of high-needs communities. Participants commonly suggested that a revised program employing bicultural workers to conduct health promotion and continue to link residents with support services would be beneficial.

### Recommendations

**Fig. 2:**
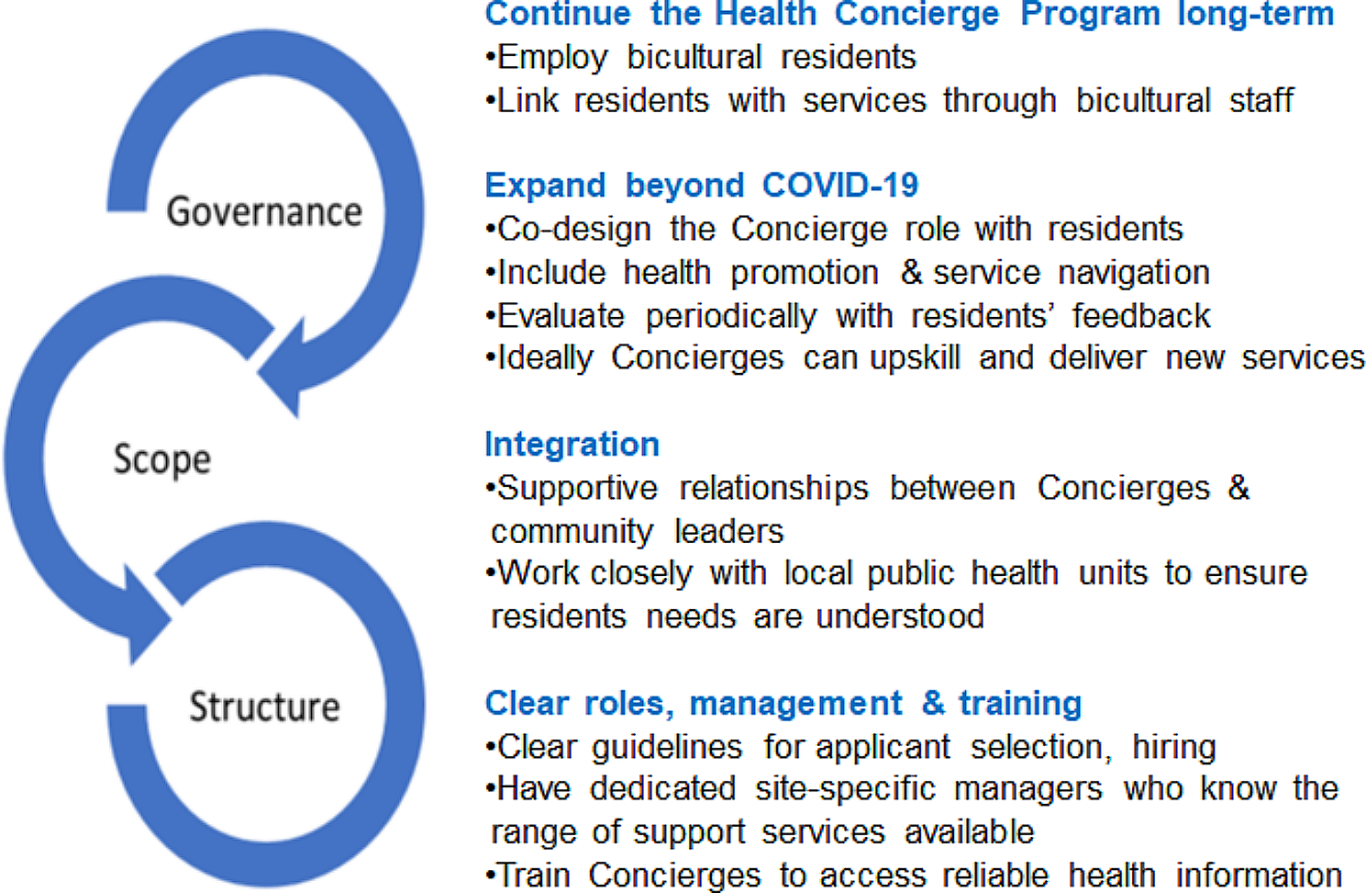
Recommendations for an extended cohealth Health Concierge Program in high-rise public housing estates

Considering our study data and advice from the PRG, we made four recommendations for a continued Program (Fig. 2).

#### Revise and continue the program long-term

There is a clear need for two-way communication and engagement in Melbourne’s multicultural public housing estates. The program brought together residents, Government and services with bicultural staff. A strength was the program’s ability to facilitate residents’ access to qualified health professionals, and disseminate information in multiple languages. This engagement should continue as an ongoing commitment to supporting residents, with a revised program seeking to improve residents’ access to health information and a variety of support services. Mechanisms incorporate feedback from residents should be in-built to enable program delivery and scope to be refined to address changing community needs.

#### Expand beyond COVID-19

Ultimately Concierges have a dual health promotion and service navigation role. The full scope of the role should be co-designed by residents. To justify the investment, any program extension should include planned, co-designed periodic evaluation with clear outcome measures. The impact of the role on Concierges themselves should be considered.

#### Integration

Building mutually supportive relationships between Concierges and community leaders supports residents’ service uptake. The program should work closely with local public health units (LPHUs) to ensure residents’ needs are understood and met in a timely manner, with any funding opportunities available through LPHUs optimised.

#### Clear roles, management and training

Flexible work and professional development opportunities would enable Concierge hiring to be inclusive and represent the community. Clear guidelines around applicant selection should be available. With a deep understanding of the people they serve, Concierges can advise stakeholders on community matters. Concierge selection criteria needs to include people who can support older adults and residents with complex health needs. Well-defined roles, management and training will promote staff accountability. This would be assisted by dedicated, site-specific managers who understand the range of services available to residents. We recommend Concierges be trained to access reliable health information such as *Health Translations* and *Better Health Channel*. Additional training could include first aid and English proficiency. Formal opportunities to de-brief with management should continue on the daily basis cohealth employed, as well as informal debriefing between Concierges.

Many of our (independent) recommendations are similar to those made by researchers following a survey of 865 HRAR residents [13]. These authors recommended making the health sector easier for residents to access, using assertive health outreach strategies, providing wrap-around services, and supporting service access [13]. A qualitative study wherein 19 (mostly older adult) residents of Flemington and North Melbourne estates were interviewed also recommended residents be consulted on public health policies which affect them, similar to our evaluation. That study concluded supports provided through Concierges were highly valued by residents and should be ongoing [33]. Our interview findings echo findings of a cohealth survey of 1,181 high-rise public housing residents across five Melbourne city councils in the first quarter of 2022; including estates in this evaluation. This identified residents’ top five priorities as: social activities to reconnect with the community; addressing mental health concerns; addressing other health concerns; gaining employment and financial security; and improving their health and fitness [34]. It is possible Concierges helped some residents address these priorities through providing information about services and organising activities.

### Public health context

In Australia, as of June 2022, people born overseas were two-times more likely die from COVID-19, in part, is due to lower COVID-19 vaccine uptake [35]. The need to increase CALD groups’ trust in COVID-19 vaccines was apparent from the start of the vaccine roll out in early 2021 [36, 37]. 

Similar to the Concierge Program, CHWs have been widely engaged in the COVID-19 pandemic response in low-middle income countries and in vulnerable communities in high-income countries [38–55]. During the pandemic, CHWs who were trusted community members played a key role as peer-educators, including in Indigenous Australian communities [44, 47, 56]. A international review of CHWs in pandemics concludes that, if adequately resourced, CHWs are critical in mitigating harm, and can help maintain essential services [57]. There are limited empirical data on CHWs’ impact on community engagement with health services and public health activities, although a Ugandan study showed participants valued CHWs’ COVID-19 home talks more than information on the radio [42]. This illustrates the value of two-way peer-led conversations in the pandemic response.

This program model may be adapted to disseminate health information in other geographically-concentrated priority groups. Groups whose trust in public authorities has been eroded are particularly at-risk from misinformation [58]. Public trust was a key factor in the ultimate success of the Victorian pandemic response– not only in the official public health messaging, but also in translations of those messages [59]. Peer-led information dissemination programs therefore have potential to overcome mistrust of authorities. Potentially a more timely outbreak response using a co-designed community participation model, such as this program, could have addressed increasing local COVID-19 transmission and averted the hard lockdown of July 2020.

In July 2022, $8.5 million in funding for a new community health program was announced by the Victorian Government. This program is called ‘Community Connectors’ and is funded for 12 months (to June 2023) in high-rise public housing estates through Homes Victoria. Community Connectors’ goal is to improve health and well-being by employing local residents to deliver targeted health promotion and connection services [60]. (*Source: Private communication from cohealth addressed to Jane Oliver 28 July 2022)*. Through our engagement with cohealth and government stakeholders, this evaluation helped shape the Community Connectors program.

### Strengths and limitations

A strength of this evaluation is its co-design aspect, ensuring our recommendations are relevant and acceptable to end-users. Further strengths include the breadth of views held by interviewed stakeholders, and the quantity and detail of the quantitative data.

We were unable to establish a direct quantifiable association between the Concierges’ work and outcomes. Concierges may have had important roles increasing COVID-19 vaccine confidence, however the drivers of vaccine uptake are complex and multifactorial [61]. Other impacts, such as some Concierges receiving their first employment in Australia, were not captured. Although Concierges were involved in multiple outbreak responses in 2021, quantitative data on their contributions were not available. Despite this, indirect evidence of impact was apparent in all the data sources we analysed. We did not receive Interaction Form data for the initial 10-months of program implementation, so are unable to examine temporal changes in the types of supports provided and residents’ engagement with Concierges. Furthermore, we note that some Interaction Form fields lacked detail, and residents reporting their satisfaction scores to Concierges may well have introduced positive response bias. As we used convenience sampling, participants may not be representative of the entire resident population. The low (38%) resident interview response rate may also have introduced bias. While thematic saturation was not noted when analysing resident interviews, this was noted among all other stakeholder groups. This, and the relatively small sample of 14 residents (most of whom were interviewed in English by a non-community member) is a limitation. Due to resource constraints, a single researcher (JO) performed the interview data analysis. Reliance on a single person’s interpretation may have affected our findings, however guidance was provided by the Evaluation Team and the PRG.

## Conclusions

The cohealth Health Concierge program that operated in Melbourne, Australia from July 2020 to 30 June 2022 rapidly disseminated up-to-date information and increased access to services for largely CALD communities during a rapidly changing pandemic response. Concierges may have contributed to high community uptake of COVID-19 testing and vaccination. CHW models such as this have potential to inform and reassure end-users as long as there is a mechanism to ensure the program can remains responsive to end-users needs. Furthermore, such models may promote protective behaviours in pandemic and non-pandemic settings, with CHWs benefiting from regular public health updates and ongoing supervision.

### Electronic supplementary material

Below is the link to the electronic supplementary material.

**Supplementary Material 1: Appendix 1:** Health Concierge Form 2.0

**Supplementary Material 2: Appendix 2:** Survey

**Supplementary Material 3: Appendix 3:** Resident Interview Guide

**Supplementary Material 4: Appendix 4.** cohealth Health Concierge Interview Guide

**Supplementary Material 5: Appendix 5.** cohealth Staff Interview Guide

**Supplementary Material 6: Appendix 6.** Interview Guide for other stakeholders

**Supplementary Material 7: Supplementary Table 1.** Ordinal regression identifying factors that predict a one-category increase in score assigned

**Supplementary Material 8: Figure S1.** Key Dates

**Supplementary Material 9: Figure S2.** Locations of the included states, Melbourne, Victoria, Australia

## Data Availability

The datasets generated during and analysed for this study are not publicly available due to privacy restrictions.
