# Supplementary material for: Evaluating a peer-to-peer health education program in Australian public housing communities during the COVID-19 pandemic
Source: BMC Health Serv Res. 2024 Feb 27;24:250. doi: 10.1186/s12913-024-10627-7 (PMC10900559; doi:10.1186/s12913-024-10627-7)
Supplement: Supplementary file 1 — Supplementary Material 1: Appendix 1: Health Concierge Form 2.0 [file 12913_2024_10627_MOESM1_ESM.pdf]

Health Concierge Form 2.0

Complete this form at the conclusion of every interaction with a resident

* Required

Health Concierge Engagement Report

Please answer all mandatory questions

1. Date? *

Format: M/d/yyyy

2. Select the suburb? *

- ◯ Footscray
- ◯ Kensington
- ◯ Collingwood
- ◯ Fitzroy
- ◯ Carlton
- ◯ North Melbourne
- ◯ Flemington
- ◯ Williamstown

3. Flemington Address? *

- ◯ 126 Racecourse Rd - Flemington
- ◯ 120 Racecourse Rd - Flemington
- ◯ 130 Racecourse Rd - Flemington
- ◯ 29 Crown Street - Flemington
- ◯ 12 Holland St - Flemington

4. North Melbourne Address? *

- ◯ 33 Alfred St - North Melbourne
- ◯ 12 Sutton St - North Melbourne
- ◯ 76 Canning St - North Melbourne
- ◯ 9 Pampas - North Melbourne
- ◯ 159 Melrose St - North Melbourne

5. Kensington Address? *

- ◯ 56 Derby St - Kensington
- ◯ 94 Ormond St - Kensington

6. Footscray Address? *

- ◯ 127 Gordon Street - Footscray

7. Fitzroy Address? *

- ◯ 90 Brunswick - Fitzroy
- ◯ 140 Brunswick - Fitzroy
- ◯ 95 Napier - Fitzroy
- ◯ 125 Napier - Fitzroy

8. Collingwood Address? *

- ◯ 253 Hoddle - Collingwood
- ◯ 229 Hoddle - Collingwood
- ◯ 240 Wellington - Collingwood

9. Carlton Address? *

- ◯ 480 Lygon - Carlton
- ◯ 478 Drummond - Carlton
- ◯ 510 Lygon - Carlton
- ◯ 530 Lygon - Carlton
- ◯ 495 Cardigan - Carlton
- ◯ 38 Elgin - Carlton
- ◯ 522 Drummond St - Carlton

10. Williamstown Address? *

- ◯ 235 Nelson Place - Williamstown
- ◯ 65 Hanmer street - Williamstown

11. What kind of engagement was provided? *

Select all that apply

- [ ] Mask distribution
- [ ] Housing support
- [ ] Hygiene and sanitising items
- [ ] Well-being / Social check-in
- [ ] Booking for a COVID-19 test
- [ ] COVID-19 related information
- [ ] Mental health information
- [ ] Other health related information
- [ ] Education support information
- [ ] Financial support information
- [ ] Vaccine related information
- [ ] Book Vaccine appointments
- [ ] ____________

Other

12. Is there further follow-up required? *

Further follow-up required by...

- ○ DHHS follow up
- ○ cohealth follow up
- ○ DHHS and cohealth follow up
- ○ I have provided the referral
- ○ No

13. Contact details for follow up

Resident Name, Phone Contact, and Address

14. Please provide details on the support given

E.g. Broken taps, Provided contact details for Mental Health programs, etc.

15. Does the resident require translated information and/or language support? *

- ○ Yes
- ○ No

16. What is the resident's preferred or primary language?

- ◯ English
- ◯ Traditional Chinese
- ◯ Simplified Chinese
- ◯ Cantonese
- ◯ Arabic
- ◯ Oromo
- ◯ Somali
- ◯ Amharic
- ◯ Tigrinya
- ◯ Dinka
- ◯ Vietnamese
- ◯ Turkish
- ◯ Indonesian
- ◯ Lao
- ◯ Polish
- ◯ Italian
- ◯ Spanish
- ◯ German

17. How useful did the resident find the health concierge service provided?

Ask the resident at the end of the interaction

	1	2	3	4	5	6	7	8	9	10	
Not at all Useful	◯	◯	◯	◯	◯	◯	◯	◯	◯	◯	Extremely Useful

18. How likely is the resident to recommend health concierge to friends or family?

Ask the resident at the end of the interaction

	1	2	3	4	5	6	7	8	9	10	
Extremely Unlikely	○	○	○	○	○	○	○	○	○	○	Extremely Likely

19. What reason(s), if any, did the resident provide for their score?

This content is neither created nor endorsed by Microsoft. The data you submit will be sent to the form owner.

Microsoft Forms
