# Supplementary material for: Evaluating a peer-to-peer health education program in Australian public housing communities during the COVID-19 pandemic
Source: BMC Health Serv Res. 2024 Feb 27;24:250. doi: 10.1186/s12913-024-10627-7 (PMC10900559; doi:10.1186/s12913-024-10627-7)
Supplement: Supplementary file 2 — Supplementary Material 2: Appendix 2: Survey [file 12913_2024_10627_MOESM2_ESM.pdf]

Survey

Please complete the survey below.

Thank you!

Hello! We are conducting a survey asking about your experiences with the cohealth Health Concierge program. Health Concierges have been working in this community. They are identifiable by their dark blue shirts with the cohealth logo.

You can skip questions or stop doing the survey at any time. Your answers will be kept strictly confidential by the research team.

First, please can you tell us a bit about about yourself

How old are you?

____
(Enter your age in years or leave blank if you prefer not to say)

What is your gender?

◯ Female
◯ Male
◯ Other
◯ Prefer not to say

What language do you mostly speak at home?

____
(Leave blank if you prefer not to say)

How many people usually live in your household?

____
(eg. if you live with 3 other people, write 4 or leave blank if you prefer not to say)

Have you spoken to a cohealth Health Concierge in your community in the last six months?

◯ Yes
◯ No
◯ Unsure
◯ Prefer not to say

How many times have you spoken to a Health Concierge in your community in the last six months?

◯ Once or twice
◯ Three to five times
◯ Six or more times

When answering the questions below, please have your experience with the Health Concierges in your community in mind.

Thinking about ALL of the interactions you've had with the Health Concierges in the last 6 months,
please state how often you felt this way by responding 'never', 'some of the time', 'most of the time', 'all of the time' to each of the questions

---

Health care competence

How often have you felt a Health Concierge was telling you everything you needed to know about your health-related problems?

◯ Never
◯ Some of the time
◯ Most of the time
◯ All of the time
◯ Prefer not to answer

How often have you felt a Health Concierge knew as much as they should about a health topic?	◯ Never<br>◯ Some of the time<br>◯ Most of the time<br>◯ All of the time<br>◯ Prefer not to answer
How often has a Health Concierge taken enough time with you during visits?	◯ Never<br>◯ Some of the time<br>◯ Most of the time<br>◯ All of the time<br>◯ Prefer not to answer
How often has a Health Concierge kept what you discussed confidential/private from others in your community?	◯ Never<br>◯ Some of the time<br>◯ Most of the time<br>◯ All of the time<br>◯ Prefer not to answer
In your opinion, how often has the Health Concierge been committed to providing the best care possible?	◯ Never<br>◯ Some of the time<br>◯ Most of the time<br>◯ All of the time<br>◯ Prefer not to answer
Respectful communication	
How often has a Health Concierge been an excellent listener?	◯ Never<br>◯ Some of the time<br>◯ Most of the time<br>◯ All of the time<br>◯ Prefer not to answer
How often have you felt better (emotionally) after seeing/talking to a Health Concierge?	◯ Never<br>◯ Some of the time<br>◯ Most of the time<br>◯ All of the time<br>◯ Prefer not to answer
How often has a Health Concierge treated you with respect?	◯ Never<br>◯ Some of the time<br>◯ Most of the time<br>◯ All of the time<br>◯ Prefer not to answer
How often has a Health Concierge had your best interest at heart?	◯ Never<br>◯ Some of the time<br>◯ Most of the time<br>◯ All of the time<br>◯ Prefer not to answer
How often has a Health Concierge made you feel that you were worthy of his/her time and effort?	◯ Never<br>◯ Some of the time<br>◯ Most of the time<br>◯ All of the time<br>◯ Prefer not to answer

Have you spoken to any other Community Health Worker (not a Health Concierge) in the last 6 months?	◯ Yes<br>◯ No<br>◯ Unsure<br>◯ Prefer not to answer
How many times have you spoken to a Community Health Worker in the last six months?	◯ Once or twice<br>◯ Three to five times<br>◯ Six or more times<br>◯ Prefer not to answer
What agency/agencies were the Community Health Worker/s from?	__________________________________

Next, we'd like to know a bit about how you feel about public health responses to infectious disease outbreaks. When answering the following questions, imagine an infectious disease outbreak were to occur in your community.

How confident are you that the Victorian public health system can respond EFFECTIVELY to protect the health of the people in your community?	◯ Very confident<br>◯ Somewhat confident<br>◯ Not too confident<br>◯ Not at all confident<br>◯ Prefer not to answer
How confident are you that the Victorian public health system will respond FAIRLY to your health needs, regardless of your race, ethnicity, income or other personal characteristics?	◯ Very confident<br>◯ Somewhat confident<br>◯ Not too confident<br>◯ Not at all confident<br>◯ Prefer not to answer
How confident are you that the Victorian public health system will provide HONEST information to the people in your community?	◯ Very confident<br>◯ Somewhat confident<br>◯ Not too confident<br>◯ Not at all confident<br>◯ Prefer not to answer
If there were an infectious disease outbreak and the Victorian public health system needed to collect information on you, such as your race, income and citizenship, how confident are you that this information would NOT be used against you?	◯ Very confident<br>◯ Somewhat confident<br>◯ Not too confident<br>◯ Not at all confident<br>◯ Prefer not to answer

Finally, we'd like to ask a few questions about your COVID-19 experience

Have you ever received information from a Health Concierge in your community about COVID-19 testing?	◯ Yes<br>◯ No<br>◯ Unsure<br>◯ Prefer not to say
Did you think this information was accurate?	◯ Yes<br>◯ No<br>◯ Unsure<br>◯ Prefer not to answer
Have you received information from the Health Concierge about COVID-19 vaccines?	◯ Yes<br>◯ No<br>◯ Unsure<br>◯ Prefer not to answer

Did you think this information was accurate?	◯ Yes<br>◯ No<br>◯ Unsure<br>◯ Prefer not to answer
Have you been tested for COVID-19 (i.e. had a swab test)?	◯ Yes<br>◯ No<br>◯ Unsure<br>◯ Prefer not to say
How many times have you been tested?	◯ 1<br>◯ 2-5<br>◯ 6-10<br>◯ More than 10<br>◯ Unsure<br>◯ Prefer not to answer
Have you been vaccinated for COVID-19?	◯ Yes<br>◯ No<br>◯ Unsure<br>◯ Prefer not to answer
Do you know where to go to get a COVID-19 vaccine for yourself?	◯ Yes<br>◯ No<br>◯ Prefer not to answer
How much do you trust the COVID-19 vaccine?	◯ Not at all<br>◯ A little<br>◯ Moderately<br>◯ Very much<br>◯ Prefer not to answer
How important do you think getting a COVID-19 vaccine is for your health?	◯ Not at all<br>◯ A little<br>◯ Moderately<br>◯ Very much<br>◯ Prefer not to say
How safe do you think a COVID-19 vaccine is for you?	◯ Not at all<br>◯ A little<br>◯ Moderately<br>◯ Very safe<br>◯ Prefer not to answer
If you don't mind letting us know, how many COVID-19 vaccine doses have you had?	◯ None<br>◯ 1 dose<br>◯ 2 doses<br>◯ 3 doses<br>◯ Prefer not to say
What was the date you had your last COVID-19 vaccine?	_________________________<br>(If unsure or you would prefer not to answer, just leave this blank)

Do you grant the researchers permission to access your COVID-19 vaccine record through the Australian Immunisation Register?<br><br>Please note this is optional - you can decline if you wish. If you choose to grant permission, we will need to collect your name, date of birth and postcode. This data is strictly confidential and will be held securely by the researchers. It won't be linked to any other information about you and will only be used to look at community vaccine uptake rates.	◯ No<br>◯ Yes
Please enter your first name	__________________________________
Please enter your last name	__________________________________
Please enter your date of birth	__________________________________
Please enter your postcode	__________________________________
