# Supplementary material for: Evaluating a peer-to-peer health education program in Australian public housing communities during the COVID-19 pandemic
Source: BMC Health Serv Res. 2024 Feb 27;24:250. doi: 10.1186/s12913-024-10627-7 (PMC10900559; doi:10.1186/s12913-024-10627-7)
Supplement: Supplementary file 3 — Supplementary Material 3: Appendix 3: Resident Interview Guide [file 12913_2024_10627_MOESM3_ESM.pdf]

Resident Interview Guide

BEFORE THE INTERVIEW:

Check that Jane has sent the info sheet and you have 2 recording devices charged up and ready to go.

PRELIMINARY DISCUSSION

"Hello, my name is ____________. I work for the University of Melbourne.

Thank you very much for agreeing to be interviewed today.

Everything that you say to me during this interview is confidential. Your privacy will be protected and you will not be identifiable to anyone outside the study team.

Most interviews take around 20 minutes.

You can stop doing the interview at any time, and if you would like to skip a question, just let me know.

You will be emailed a $50 Coles Supermarket voucher as a thanks for your time being interviewed.

Can I just check that your email address is: *(read it out and confirm, if they don't want it emailed then get their address and I'll mail it out)*

Do you have any questions for me before we start?"

"Are you happy for me to start recording now?"

If Yes, start recording on BOTH devices. If No, the interview cannot proceed.

INTERVIEW QUESTIONS

- First would you mind telling me a bit about yourself, like how long you've lived in (*name community)* and what languages you speak at home.

Health Concierges are the people in blue cohealth shirts who sit in the foyer of your building. My questions are mostly about them.

- Have you seen a Health Concierge in your building in the last six months?
- If yes, have you spoken to a Health Concierge in your building?
    - If yes, how many times in the last six months?
    - Did you talk to the Health Concierge about COVID-19?

*If **NO**: They did not talk to the Health Concierge about COVID-19*

- Would you feel comfortable speaking to the Health Concierge? Why/ why not?
- Do you feel that the information they give people is trustworthy?

- Why or why not?
- Have you found information from the Health Concierge to be helpful at all? Why or why not?

*If **YES**: They talked to the Health Concierge about COVID-19*

- Did you feel comfortable speaking to the Health Concierge about COVID-19? Why or why not?
- Can you let me know just generally what sorts of things you talked about with them?
- Did they give you information about COVID testing at all?
  - If yes, did you feel like this information was trustworthy? Why or why not?
  - Did you find this information helpful? Why or why not?
  - Do you think receiving this information made you more likely to get tested? Why or why not?

- Did they give you information about COVID-19 vaccines?
  - If yes, did you feel like this information was trustworthy? Why or why not?
  - Did you find this information helpful? Why or why not?
  - Do you think receiving this information made you more likely to be vaccinated? Why or why not?
  - Do you think receiving this information made you more likely to want to get a COVID-19 vaccine booster shot? Why or why not?
  - Do you think people would talk to the HC if they had questions about COVID vaccines, like whether they should get them for their children?
  - Do you mind if I ask, do you have children? If YES: Did speaking with the HC make you more or less likely to want your children to get the COVID-19 vaccine?

- Can I ask, have you had any COVID vaccines?
  Why/why not?
  How many have you had? Do you think you'll get the third vaccine? Why/why not?

- Have you received information from the Health Concierge about anything else to do with COVID-19?
  - If yes, did you feel like this information was trustworthy?
  - Why or why not?
  - Did you find this information helpful? Why or why not?
  - Do you think receiving this information made you more likely to follow the COVID rules (like social distancing, wearing masks)? Why or why not?

- If you had a question about COVID or something to do with COVID, do you think you would ask the Health Concierge?

- How likely would you be to ask other cohealth staff about that? (ie. not a Health Concierge, a cohealth nurse for example)
- How likely would you be to ask Government staff, like calling someone from the Department of Health?

- If your family member or friend had a question about COVID, would you recommend they ask a Health Concierge?

Other

- If you had a personal problem with your health, how likely would you be to discuss this with a Health Concierge in your building?
- Is there any way you think what the Health Concierges do could be improved?
- Apart from providing COVID services, how else might the Health Concierges be able to help the community?
- Can you think of any other services that your community would benefit from having on-site?
- Is there anything else you think I should know that we haven't talked about?

That's the end of my questions for you today.

Thank you very much for your help with this study, it is appreciated.

My supervisor, Jane Oliver, will send you a $50 Coles supermarket voucher.

Please contact her if it does not arrive. Her contact information is on the study information sheet.

Do you have any questions for me before I go?

Thank you. Have a nice day.
