# Supplementary material for: Evaluating a peer-to-peer health education program in Australian public housing communities during the COVID-19 pandemic
Source: BMC Health Serv Res. 2024 Feb 27;24:250. doi: 10.1186/s12913-024-10627-7 (PMC10900559; doi:10.1186/s12913-024-10627-7)
Supplement: Supplementary file 4 — Supplementary Material 4: Appendix 4. cohealth Health Concierge Interview Guide [file 12913_2024_10627_MOESM4_ESM.pdf]

Appendix 4. cohealth Health Concierge Interview Guide

Background

- How long have you been/were you working with cohealth? Would you mind sharing your age, gender, and what languages you speak at home?
- What is/was your role with cohealth? How long have you been/were you in this position?
- What's been your experience with cohealth's COVID-19 responses in the high-rise towers?
- What's been your experience with cohealth's Health Concierge program?
- Can you tell me a bit about the aims of the HC program, and how it works to reach those aims?

Health Concierge selection and training

- Can you tell me about how you started working with cohealth? Why did you decide to work with them?
- Can you tell me about the training you received about COVID-19?
    - Prompts: testing, vaccination
    - Was this information useful? Why/why not?
    - Was there information you think you should have received but didn't?
- Do you feel you have a good understanding of COVID-19 testing?
- Do you feel you have a good understanding of COVID-19 vaccination?

Provision of information to residents

- Can you tell me about how you provide information to residents?
    - Prompts: walk-up appointments, scheduled appointments, written information, electronic information
- Do you assist lots of different people, or is it usually the same people?
- How often do you talk to residents about COVID-19 as opposed to other topics?
- What other topics have you discussed with residents?
- Do you feel comfortable providing COVID-19 information to residents? Why/why not?
- Do you think the residents of the high-rise towers are aware they can access COVID-19 related information through the Health Concierge? Why or why not?
- Do you think the residents of the high-rise towers are accessing COVID-19 related information through the Health Concierge? Why or why not?
- Do you think the residents of the high-rise towers are accessing COVID-19 related information through other sources? If so, what sources?

Residents' trust in the Health Concierge program

- Do you think the Health Concierge program has made a difference in residents' trust in and uptake of COVID-19 testing? How so/why not?
- Do you think the Health Concierge program has made a difference in residents' trust in and uptake of COVID-19 vaccination? How so/why not?
    - How do you see work in this area continuing, eg. COVID-19 vaccine uptake for children, boosters?

Health interventions and the role of the Health Concierge program

- Can you tell me about some of the public health interventions undertaken in the towers as part of the COVID-19 response?
- Do you feel you have a good understanding of the health interventions in the towers and why they've been undertaken?
- What has been the role of the Health Concierges in the interventions?
- Have you provided cohealth with information that helped inform the interventions? Can you describe this to me, in general terms? Why did you decide to provide cohealth with this information? *Prompt: Do you trust cohealth with this information?*
- Have you provided residents with information about the interventions? What information was this, and how was it received?
- Do you feel comfortable providing residents with information about the interventions? Why/why not?
- Do you feel residents' relationship with DH-instigated public health activities has changed for better or worse since the Health Concierge program started? Why?
- How likely are residents to want to engage with public health activities? What are their key barriers to engaging and how could these be overcome?
- Do you feel that the Health Concierge program has made it easier for residents to engage with public health activities? Why?

Operations of the Health Concierge program

- How have residents' needs for information and support around COVID-19 changed through the course of the pandemic?
- Has cohealth been able to respond proactively to the changing needs of residents during the pandemic? How has this happened, or why not?
- Is cohealth able to match the demand for the Health Concierge program? Why or why not?
- Do you think the residents' needs are met by the Health Concierge program? How so/why not?
- Has information and feedback coming from the HCs or other community representative lead to HC program refinements? How responsive has the HC program been to community feedback?
- How could the Health Concierge program be expanded to support improving health outcomes for public housing residents?

Impact on Health Concierges

- What challenges do you face in your role as a Health Concierge, and how could these be overcome?
- How could Health Concierges be better supported to optimise health promotion in their communities?
- What resources, including training, financial investments and partnership development, need to be instituted in order to ensure sustainability of the 'Health Concierge' program?
- Do you feel being a Health Concierge has helped your employment opportunities?
- Do you feel you have job security as a Health Concierge? Why or why not?

Other

- Is there any way you think the Health Concierge program could be improved?
- Is there anything else you think I should know that we haven’t talked about?
- Can you recommend anyone else for us to speak to about the Health Concierge program?
