# Supplementary material for: Evaluating a peer-to-peer health education program in Australian public housing communities during the COVID-19 pandemic
Source: BMC Health Serv Res. 2024 Feb 27;24:250. doi: 10.1186/s12913-024-10627-7 (PMC10900559; doi:10.1186/s12913-024-10627-7)
Supplement: Supplementary file 6 — Supplementary Material 6: Appendix 6. Interview Guide for other stakeholders [file 12913_2024_10627_MOESM6_ESM.pdf]

Appendix 6. Interview Guide for other stakeholders

Background

- Would you mind sharing your age, gender, and what languages you speak at home?
- What role do you work in?
- How long have you been in this position?
- What's been your experience with cohealth's COVID-19 responses in the high-rise towers?
- What's been your experience with cohealth's Health Concierge program?
- Can you tell me a bit about the aims of the HC program, and how it works to reach those aims?

Health Concierge selection and training

- Have you had much to do with the Health Concierges?
- What do you think of the job they are doing?

Provision of information to residents

- Do you think residents of the high-rise towers benefit from the Health Concierge service? Why or why not?
- Do you think the residents are aware they can access information through the Health Concierge? Why or why not?
- Do you think the residents of the high-rise towers are provided with information through the Health Concierge? Why or why not? What sort of information are the residents getting?

Residents' trust in the Health Concierge program

- Do you think the Health Concierge program has made a difference in residents' trust in and uptake of COVID-19 testing? How so/why not?
- Do you think the Health Concierge program has made a difference in residents' trust in and uptake of COVID-19 vaccines? How so/why not?

Health interventions and the role of the Health Concierge program

- Can you tell me about some of the public health interventions undertaken in the towers as part of the COVID-19 response?
- What has been the role of the Health Concierges in the interventions?
- Have the Health Concierges provided information that helped inform the interventions? Can you describe this to me, in general terms?
- Have the Health Concierges provided residents with information about the interventions? What information was this, and how was it received?
- Do you feel residents' relationship with government instigated public health activities has changed for better or worse since the Health Concierge program was instigated? Why?
- How likely are residents to want to engage with public health activities? What are their key barriers to engaging and how could these be overcome?
- Do you feel that the Health Concierge program has made it easier for residents to engage with public health activities? Why?

Operations of the Health Concierge program

- How have residents' needs changed through the course of the pandemic?
- Has cohealth been able to respond proactively to the changing needs of residents during the pandemic? How has this happened, or why not?
- Is cohealth able to match the demand for the Health Concierge program? Why or why not?
- Do you think the residents' needs are met by the Health Concierge program? How so/why not?
- How sustainable do you feel the Health Concierge program is? How could sustainability be optimised?
- How could the Health Concierge program be expanded to support improving health outcomes for public housing residents?
- Do you think the HC program has had an impact beyond COVID-19? If so, how?
- Do you think the HC program has a role beyond the COVID-19? If so, how?

Impact on Health Concierges

- How could Health Concierges be better supported to optimise health promotion in their communities?
- What resources, including training, financial investments and partnership development, need to be instituted in order to ensure sustainability of the 'Health Concierge' program?

Other

- Is there any way you think the Health Concierge program could be improved?
- Is there anything else you think I should know that we haven't talked about?
  Can you recommend anyone else for us to speak to about the Health Concierge program?
