# Supplementary material for: Evaluating a peer-to-peer health education program in Australian public housing communities during the COVID-19 pandemic
Source: BMC Health Serv Res. 2024 Feb 27;24:250. doi: 10.1186/s12913-024-10627-7 (PMC10900559; doi:10.1186/s12913-024-10627-7)
Supplement: Supplementary file 7 — Supplementary Material 7: Supplementary Table 1. Ordinal regression identifying factors that predict a one-category increase in score assigned [file 12913_2024_10627_MOESM7_ESM.docx]

**Supplementary Table 1. Ordinal regression identifying factors that predict a one-category increase in score assigned**

| **Factor** | **Health Concierge healthcare competence score**  **(95% confidence interval; p-value)** | **Health Concierge respectful communication score**  **(95% confidence interval; p-value)** | **Trust in public health authorities score**  **(95% confidence interval; p-value)** |
| --- | --- | --- | --- |
| **Language mostly spoken at home**  English  Other | Reference  0.79 (0.37-1.69; p=0.54) | Reference  1.01 (0.45-2.27; p=0.97) | Reference  **2.74 (1.49-5.07; p<0.01)** |
| **Age (years)** | 1.00 (0.98-1.02; p=0.97) | 1.00 (0.98-1.01; 0.72) | 1.00 (0.99-1.02; p=0.64) |
| **Sex**  Male  Female | Reference  1.19 (0.63-2.26; 0.59) | Reference  0.82 (0.41-1.67; p=0.59) | Reference  1.61 (0.93-2.78; 0.09) |
| **Experienced Operation Benessere**  No  Yes | Reference  1.00 (0.57-1.77; p=0.99) | Reference  1.14 (0.62-2.12; p=0.67) | Reference  0.88 (0.53-1.48; p=0.64) |
| **COVID-19 vaccination status**  <2 doses  ≥2 doses | Reference  1.42 (0.35-5.83; p=0.63) | Reference  1.28 (0.34-4.88; p=0.71) | Reference  **5.27 (1.89-14.70; p<0.01)** |
| **No. of COVID-19 tests undertaken**  Five or fewer tests  >5 tests | Reference  **2.47 (1.35-4.51; p<0.01)** | Reference  1.26 (0.67-2.36; p=0.47) | Reference  1.26 (0.72-2.21; p=0.41) |
| **Trust in public health authorities score**  Not at all  A little  Moderate  High | Reference  3.07 (0.25-37.80; p=0.38)  4.55 (0.40-51.30; p=0.22)  4.71 (0.43-52.00; p=0.21) | Reference  1.86 (0.16-20.90; p=0.62)  2.86 (0.28-29.90; p=0.38)  3.27 (0.33-52.00; p=31.00) |  |

*Bold typeface indicate the finding is statically significant*
