# Supplementary figures and images for: Evaluating a peer-to-peer health education program in Australian public housing communities during the COVID-19 pandemic
Source: BMC Health Serv Res. 2024 Feb 27;24:250. doi: 10.1186/s12913-024-10627-7 (PMC10900559; doi:10.1186/s12913-024-10627-7)

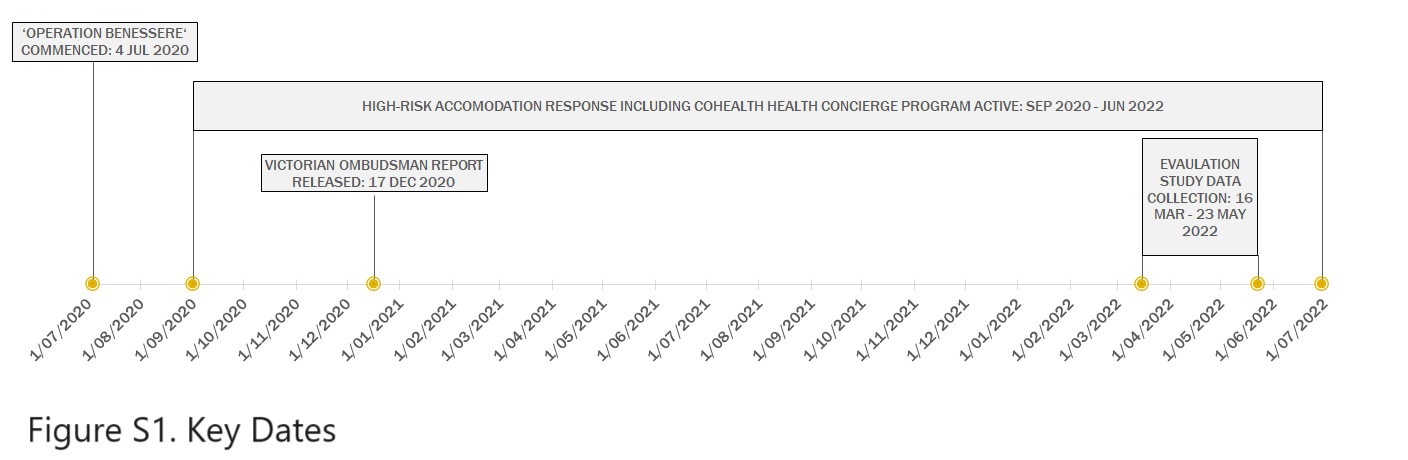

Supplement: Supplementary file 8 — Supplementary Material 8: Figure S1. Key Dates [file 12913_2024_10627_MOESM8_ESM.png]

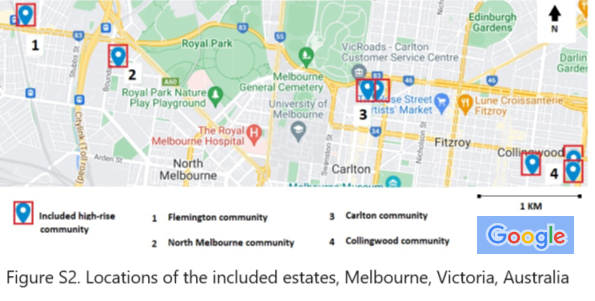

Supplement: Supplementary file 9 — Supplementary Material 9: Figure S2. Locations of the included states, Melbourne, Victoria, Australia [file 12913_2024_10627_MOESM9_ESM.png]
